# Stereotactic Radiotherapy for Ultra-Central Lung Oligometastases in Non-Small-Cell Lung Cancer

**DOI:** 10.3390/cancers12040885

**Published:** 2020-04-05

**Authors:** Mauro Loi, Davide Franceschini, Luca Dominici, Ciro Franzese, Ilaria Chiola, Tiziana Comito, Marco Marzo, Giacomo Reggiori, Pietro Mancosu, Stefano Tomatis, Joost Nuyttens, Marta Scorsetti

**Affiliations:** 1 Radiotherapy and Radiosurgery Department, Humanitas Cancer Center, 20089 Rozzano, Milan Area, Italy; 2 Radiotherapy Department, University of Florence, 50100 Florence, Italy; 3 Radiotherapy Department, Erasmus MC Cancer Center, 3015GD Rotterdam, The Netherlands

**Keywords:** non-small-cell lung cancer, ultra-central, stereotactic radiotherapy, oligometastases, biologically effective dose

## Abstract

Background: Stereotactic body radiotherapy (SBRT) in ultra-central (UC) lung tumors, defined in the presence of planning target volume (PTV) overlap or direct tumor abutment to the central bronchial tree or esophagus, may be correlated to a higher incidence of severe adverse events. Outcome and toxicity in oligometastatic (≤3 metastases) non-small-cell lung cancer (NSCLC) patients receiving SBRT for UC tumors were evaluated. Methods: Oligometastatic NSCLC patients treated with SBRT for UC were retrospectively reviewed. Local control (LC), distant metastasis-free survival (DMFS), progression-free survival (PFS) and overall survival (OS) were calculated. Incidence and grade of toxicity were evaluated. Statistical analysis was performed to assess the impact of clinical and treatment-related variables on outcome and toxicity occurrence. Results: Seventy-two patients were treated to a median biologically effective dose (BED) of 105 (75–132) Gy^10^. Two-year LC, DMFS, PFS, and OS were 83%, 46%, 43%, and 49%. BED>75 Gy^10^ was correlated to superior LC (*p* = 0.02), PFS (*p* = 0.036), and OS (*p* < 0.001). Grade ≥3 toxicity rate was 7%, including one fatal esophagitis. No variables were correlated to DMFS or to occurrence of overall and grade ≥3 toxicity. Conclusions: SBRT using dose-intensive schedules improves outcome in NSCLC patients. Overall toxicity is acceptable, although rare but potentially fatal toxicities may occur.

## 1. Introduction

Lung cancer is among the most frequent malignancies worldwide and is burdened by a high disease-related mortality [1]. About 85% of cases are non-small-cell lung cancer (NSCLC) [1,2]. For patients with oligometastatic disease, defined as ≤3 or fewer concurrent metastases [3], use of ablative local treatments may improve outcome: multiple treatment options have been applied in this setting, including surgery, radiofrequency, and stereotactic radiotherapy (SBRT) [4]. Surgical therapy, the treatment of reference for oligometastases in historical series, has been extensively described for adrenal, lung, and brain metastases [5,6,7,8], but its use is traditionally limited to selected candidates [7,9]. Use of radiofrequency ablation may be limited by its invasive nature, with consequent risk of complications such as pneumothorax and bleeding [10]. Due to its noninvasive character and possibility to treat multiple targets during the same treatment course, SBRT emerged in the last decade as a valuable treatment option, resulting in excellent local control rates and possible survival benefits [11,12]. However, tumor location influences the indication to treatment and the choice of dose regimen in patients eligible to SBRT: life-threatening adverse events, such as broncho-esophageal fistulae and hemorrhage, have been reported in patients undergoing SBRT on central tumors (defined as tumors located within 2 cm of the proximal bronchial tree, heart, great vessels, trachea, or other mediastinal structures), if an ablative dose is delivered in three fractions [13]. Conversely, use of more protracted dose schedules (4–12 fractions) showed an acceptable toxicity incidence with satisfying oncologic outcomes [14,15,16,17,18]. Recently, a subset of proximally-seated central tumors, dubbed “ultra-central”, has been reported as a separate clinical entity due to possible additional increased risk of fatal toxicity [19]. However there is no consensual definition of "ultra-central" tumors. Chaudhuri et al. classified central tumors as "ultra-central" if the gross tumor volume (GTV) directly invaded the proximal bronchial tree or trachea [20], and the same definition has also been used by Haseltine et al. [21]. On the other hand, Tekatli et al. defined as ultra-central those tumors for which the planning target volume (PTV) overlaps the trachea or main bronchi [22]. Recently, a more consensual definition was introduced in the prospective dose-finding SUNSET trial, enrolling patients with tumors ≤6 cm whose PTV touches or overlaps the central bronchial tree, esophagus, pulmonary vein, or pulmonary artery [23]. Finally, controversy arose about the most suitable dose and fractionation schedule for the treatment of ultra-central tumors due to stringent trade-offs between the need for an ablative dose delivery (traditionally corresponding to a biologically effective dose of 100 Gy for lung tumors [24]) and risk of radiation injury in case of over-irradiation of proximal critical structures; Tekatli et al. reported a 15% fatal toxicity rate (mainly due to pulmonary hemorrhage) in patients with ultra-central tumors who received a treatment regimen delivering 60 Gy in 12 fractions [22]. The aim of our study is to evaluate the safety and efficacy of different SBRT regimens for the treatment of ultra-central NSCLC oligometastases and to assess the predictive influence of clinical and treatment-related factors on outcome and incidence of toxicity. 

## 2. Results

### 2.1. Patient- and Treatment-related Characteristics

Patient- and treatment-related characteristics are summarized in Table 1. Median follow-up per patient from SBRT completion was 17 months (interquartile range, IQR 8–24 months). Median age at SBRT was 75 years (IQR 67–80 years). Histologic subtypes were adenocarcinoma and squamous cell carcinoma in 75% (*n* = 54) and 25% (*n* = 18) of patients, respectively. In the majority of cases (*n* = 70, 98%), the PTV encompassed the central bronchial tree, at the level of the main bronchi (*n* = 36, 50%) and the lobar bronchi (*n* = 34, 48%). The PTV–esophagus overlap occurred in two patients (2%). Systemic therapy, excluding adjuvant chemotherapy for primary cancer, was administered prior to SBRT in 27 patients (38%), consisting of 1 or ≥2 chemotherapy lines in 28% (*n* = 20) and 10% (*n* = 7) of patients, respectively. Different dose regimens were used, consisting of 50 Gy/5 fractions, 45 Gy/6 fractions, 48–60 Gy/8 fractions, and 50–70 Gy/10 fractions radiation schedules in 18%, 10%, 61%, and 11% (*n* = 13, 7, 44, 8) of patients, respectively. Median prescribed biologically effective dose (BED) was 105 Gy^10^ (range 75–132 Gy^10^).

### 2.2. Local Control

**Figure 1:**
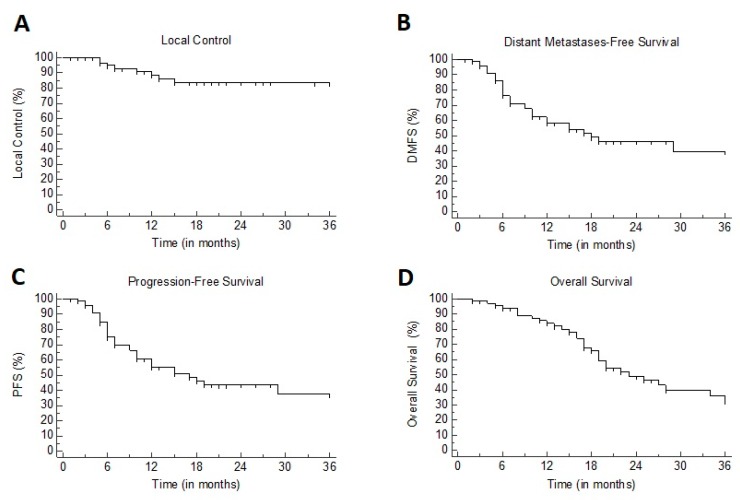
Kaplan–Meier plots for: (**A**) local control (LC). (**B**) distant metastases-free survival (DMFS). (**C**) progression-free survival (PFS). (**D**) overall survival (OS).

**Figure 2:**
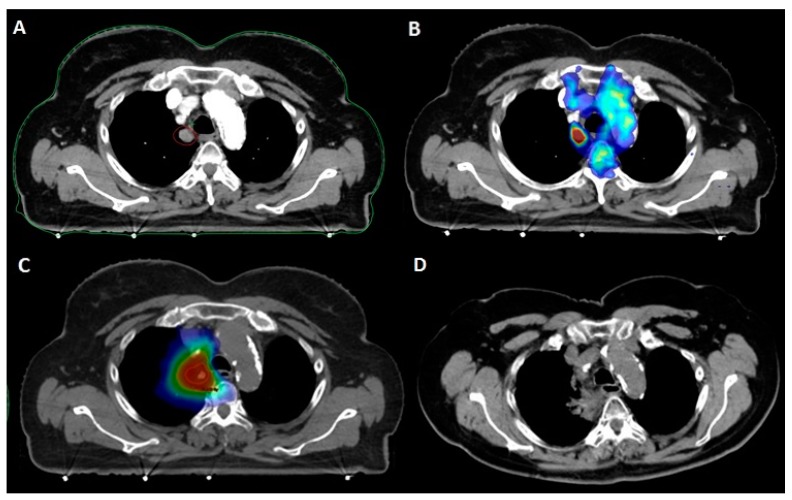
Patient with a clinical history of pT2N0 lung adenocarcinoma treated with upper lobe lobectomy. **A**: Paramediastinal middle lobe metastasis (12 × 13 mm) detected at follow-up contrast-enhanced chest CT. **B**: 18FDG-PET/CT fusion showing isolated hypermetabolism of the paramediastinal metastasis. **C**: SBRT delivering 50 Gy in five fractions to the PTV (dark blue: 20 Gy; light blue: 30 Gy; green: 40 Gy; yellow: 45 Gy; red: 50 Gy). **D**: CT evaluation at nine months showing radiation fibrosis following complete metabolic response.

At three months from SBRT, ORR was 91%, consisting of SD, PR, and CR in 27%, 30%, and 33% of patients respectively. Rates of LC were 91% at one year and 83% at two years; median not reached (Figure 1A and Figure 2). At univariate analysis, only the administration of a BED > 75 Gy^10^ was significantly associated (*p* = 0.02) with improved LC (see Table 1).

### 2.3. Progression-Free Survival

At one and two years, DMFS was 58.5% and 46%, respectively, with a median of 18 months (95% CI 10–53) (Figure 1B). At univariate analysis, patients previously treated with chemotherapy showed poorer DMFS as compared to chemotherapy-naïve patients (7 versus 44 months, *p* = 0.003). PFS rates were 57% and 43% at one and two years, respectively, with a median of 17 months (95% CI 10–46) (Figure 1C). At univariate analysis, BED >75 Gy^10^ was correlated with improved PFS (17 versus 6 months, *p* = 0.036), while prior use of chemotherapy was associated with poorer PFS (7 versus 44 months, *p* = 0.008) (Table 1). Multivariate analysis confirmed the independent prognostic impact of BED >75 Gy^10^ (hazard ratio, HR: 0.25, 95% CI 0.07–0.88; *p* = 0.03) and prior use of chemotherapy (HR 2.44, 95% CI 1.25–4.75; *p* = 0.009). 

### 2.4. Overall Survival

At one and two years, OS was 84% and 49%, respectively, with a median of 22 months (Figure 1D). At univariate analysis, BED >75 Gy^10^ correlated with improved survival: 24 versus 7 months, *p* < 0.001 (Table 1). 

### 2.5. Toxicity

**Figure 3:**
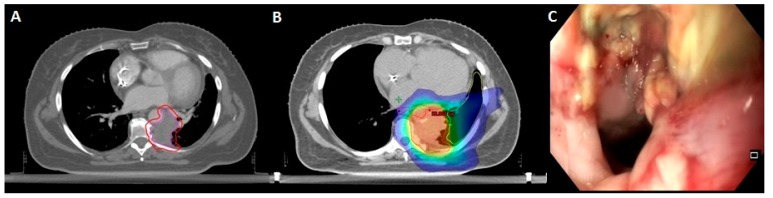
Patient with a clinical history of pT1aN0 lung adenocarcinoma treated with lingulectomy. **A**: Histologically-proven adenocarcinoma metastasis (34 mm) of the left paravertebral space, treated with SBRT (purple: GTV; red: PTV). **B**: SBRT delivering 50 Gy in five fractions to the PTV (dark blue: 20 Gy; light blue: 30 Gy; green: 40 Gy; yellow: 45 Gy; red: 50 Gy). **C**: Esophageal endoscopy following painful dysphagia and cachexia: extrinsic compression from tumor relapse associated to ulcerated mucosal ulceration possibly related to radiation treatment.

Report of toxicities is summarized in Table 2. Treatment-related adverse effects, all grade confounded, were observed in 30% of the patients: this consisted, in most cases, of mild grade 1–2 radiation pneumonitis (15%) and esophagitis (6%), as well as two cases of minor, spontaneously resolving hemoptysis. However, severe toxicity (grade ≥3) occurred in five cases, consisting mostly of radiation pneumonitis or hemoptysis requiring medical intervention and admission at the emergency department. At statistical analysis, no variable was correlated with the onset of overall or grade ≥3 toxicity (see Table 3). One patient experienced respiratory failure following stenosis of the main bronchus requiring endoscopic dilatation after fine needle aspiration excluded a disease recurrence. Finally, one possible grade 5 toxicity was reported in a 76-year-old patient with a clinical history of resected pT1a lung adenocarcinoma, who underwent SBRT (delivering 50 Gy in five fractions) to a lung metastasis located in proximity to the main left bronchus. During the following three months, she experienced a severe deterioration of her general conditions linked to painful dysphagia and cachexia ultimately leading to anorexia and death. A contrast-enhanced CT detected a massive intramediastinal relapse, resulting in a possible mass effect on the feeding tube; however, esophagogastroduodenoscopy showed an ulcerated bleeding esophageal mucosa, as well as extrinsic compression, thus a possible component related to radiation injury could not be excluded (Figure 3). 

## 3. Discussion

Here we report outcome and toxicity data on 72 patients affected by ultra-central oligometastases from NSCLC primaries. With an aggressive approach, we report excellent LC rates (91% at one year and 83% at two years), while DMFS and PFS are in line with the clinical scenario presented, confirming that distant relapse is the most common pattern of disease relapse in oligometastatic patients receiving ablative local therapy.

An increasing body of literature is now supporting the use of local ablative approaches with curative intent in oligometastatic patients, the most convincing data arising from the group of NSCLC patients [11,25,26]. Nevertheless, in clinical situations such as the occurrence of ultra-central lung oligometastases, the delivery of SBRT to radical doses can be technically challenging.

Since the pivotal trials of SBRT for lung tumors, it emerged quite clearly that the incidence of toxicity observed in cases of tumors located within 2 cm around the proximal bronchial tree was significantly higher as compared to peripheral tumors, while using the same doses and number of fractions [13]. Various attempts have been conducted to identify a less aggressive, although equally effective, stereotactic treatment regimen for central tumors, showing that the delivery of a BED > 100 Gy in five or more fractions resulted in a safe and effective treatment, with results similar to those obtained for peripheral targets [15,16,17,27,28]. However, scarce data are available concerning the treatment of ultra-central tumors, for which it is still unclear whether these fractionation schedules can be used with the same tolerance and efficacy. Among future strategies, expanding use of proton therapy and hadrons (for example, carbon ion) may allow potential dosimetric advantage over photon-based SBRT in reducing dose exposure of critical structures while maintaining target tumor dose coverage. In a recent meta-analysis by Chi et al., a lower incidence of grade ≥3 toxicity was associated with the use of particle therapy (0.9% vs. 3.4%, *p* = 0.001) [29].

In our study, although most patients were treated with a BED > 75 Gy^10^, toxicity was manageable, with only one case of suspected G5 toxicity reported and five cases of G3 toxicity fully recovered following medical treatment. In most patients, SBRT was well tolerated. Along with this, we also observed high tumor control rates, with a significant impact of delivered dose: in patients receiving a BED of 75 Gy^10^ (corresponding to 50 Gy in 10 fractions, a schedule proposed for ultra-central tumors [30]), the local control rate was significantly lower. While some authors advocate for dose de-escalation to a BED ≤ 60 Gy^10^ in ultra-central tumors in an effort to prioritize safety of the organs at risk (OARs), the use of low-dose regimens results in inferior tumor control rates, with two-year LC 59%–70% [31,32]. Our results stress the need for sufficiently intensive dose-regimens in order to warrant durable disease control. The relevance of the delivered dose can also be extrapolated by another recent series, including patients with ultra-central lung tumors treated with a median BED of 59.5 Gy. Twenty-four out of 51 patients developed a local recurrence, with a one-year local control rate of 54.4%, compared with 91% as shown in our study [33].

Most notably, we found that delivering an ablative dose to the ultra-central metastases can change the course of the disease, since the PFS was 6 times longer in patients treated with BED >75 Gy and a significant impact on OS was also identified. This observation stresses the need for an ablative treatment regimen in oligometastatic patients, even in the event of ultra-central localization. 

Our data are consistent with those published by Chang et al. in 2018. Authors evaluated 107 patients with primary and metastatic central and ultra-central tumors treated with five-fraction schedules. The author found no difference in LC, OS, and grade ≥3 toxicity comparing patients with central and ultra-central tumors [34]. A recent review analyzed data from 10 studies focusing on SBRT for ultra-central lung tumors, for a total number of 250 treated patients [19]. Despite obvious limitations linked to the retrospective nature of the study and the heterogeneity of delivered doses (BED ranging from 48 to 138 Gy), safety was confirmed. Median treatment-related grade 3 or greater toxicity was 10% (range, 0%–50%). Median treatment-related mortality rate was 5%. Authors also reported that higher doses to the proximal bronchial tree, concurrent bevacizumab use, and antiplatelet/anticoagulant use were correlated with increased toxicity. In our series, we were not able to find any significant correlation between analyzed variables and toxicity, probably due to the small study population and the low number of events that occurred. Apart from the BED, we also found prior administration of chemotherapy as a strong determinant of PFS. Indeed, chemotherapy-naïve patients had a median PFS of 44 months compared to 7 months in patients who had already received at least one chemotherapy line. Although counter-intuitive, this parameter is starting to be more and more present in the literature of oligometastatic patients, since it is a simple and immediate surrogate of the advancement of the disease. Chemotherapy-naïve patients are more frequently at an earlier stage of their disease and may exhibit a more radiosensitive phenotype in comparison to heavily-pretreated patients, resulting in longer disease remission intervals. In a large series of oligometastatic patients from different primaries and various sites, the administration of systemic therapies prior to SBRT predicted a higher risk of progression, with an HR of 1.19 [35]. Similarly, Sharma et al. reported poorer local control in patients previously treated with chemotherapy in a large database of 327 pulmonary oligometastases from miscellaneous primary tumors [36]. 

Despite the well-known limitations of a retrospective study and the heterogeneity of treatment regimens, our experience is one of the largest published series on oligometastatic ultra-central tumors by NSCLC. The results we report are very satisfactory in terms of safety and local control and confirm that ablative doses are required in the oligometastatic setting, as far as reasonably acceptable, and have a real impact on the patients‘ prognosis. 

## 4. Materials and Methods 

### 4.1. Patient Selection, Treatment Procedures, and Follow-up

Ultra-central tumors were defined as tumors for which the planning target volume(PTV) touches or overlaps the following organs at risk (OARs): central bronchial tree, esophagus, pulmonary vein, or pulmonary artery [23]. Oligometastatic disease was defined as three or fewer concurrent metastases. According to these criteria, 72 consecutive patients undergoing SBRT for ultra-central NSCLC oligometastases from February 2013 to July 2019 were identified, accounting for 72 treated tumors. For all patients, pathological confirmation with a biopsy was obtained at the diagnosis or at the first disease relapse. Indication to treatment was discussed at the Multidisciplinary Tumor Board. In all cases, a 1.5-mm-thick slice simulation 4D CT was acquired, and an internal target volume (ITV) was delineated to take into account respiratory motion of the gross tumor volume (GTV). PTV was obtained by an isotropic expansion of 5 mm of the ITV. An assessment of efficacy was performed using the revised Response Evaluation Criteria In Solid Tumors (RECIST version 1.1, 2009). Incidence of toxicity was evaluated using the Common Terminology Criteria for Adverse Events (CTCAE version 4.03, 2010). A clinical evaluation with a physical examination and CT-scan was performed every three months or in presence of clinical symptoms: in case of suspicious CT progression, additional confirmation by 18FDG-PET or targeted biopsy may be required. Informed consent was obtained by all the patients or relatives (in case of deceased patients), and investigations were conducted according to Declaration of Helsinki principles. The study was carried out according to the national regulation on biomedical research (study n°2114/ID733).

### 4.2. Definition of the Endpoints

The objective response rate (ORR) was defined as the presence of stable disease (SD), partial response (PR), or complete response (CR) at the first CT re-evaluation at three months. Local failure (LF) was defined as an increase of ≥20% in the diameter of the target lesion. Local control (LC) was calculated from SBRT completion to the date of LF or the last follow-up. Distant metastasis-free survival (DMFS) was measured from SBRT completion until the onset of distant metastases outside the irradiated area. Progression-free survival (PFS) was defined as the delay between SBRT completion and disease progression at any site. Overall survival (OS) was measured from SBRT completion until death from any cause or last follow-up. 

### 4.3. Statistical Analysis

Clinical (age, proximity OAR, histological subtype, timing of metastases from primary tumor diagnosis, tumor size) and treatment-related (prior chemotherapy, total delivered dose, overall treatment time) variables were summarized using descriptive statistics. To allow for comparison among different dose regimens, all radiation doses were expressed as biologically effective dose according to the formula total dose × [1 + (dose per fraction/(α/β))], assuming an α/β of 10 for NSCLC (BED). Survival plots were calculated using the Kaplan–Meier method. Univariate analysis using the log-rank test was performed to investigate the predictive impact of dichotomized categorical variables on outcome. The Cox multivariate model was applied in case of multiple predictive factors that were found significant at univariate analysis. The correlation between variables and overall or severe (CTCAE grade ≥ 3) toxicity was investigated using the chi^2^-test. A *p*-value ≤0.05 was considered statistically significant. Statistical analysis was performed using MEDCalc statistical software version 19.0.3 (MedCalc Software bvba, Ostend, Belgium).

## 5. Conclusions

SBRT for ultra-central lung tumors in oligometastatic NSCLC is safe, with a low incidence of severe toxicity. The delivery of ablative doses is required to avoid local progression and may change the natural history of the disease.

## Figures and Tables

**Table 1:** Clinical and treatment-related variables and impact on local control, distant metastases-free survival, progression-free survival, and overall survival. NR: not reached; BED: biologically effective dose. Italic: variable type. Bold: significant at statistical analysis (*p* < 0.05).

Clinical and Treatment-Related Variables	Local Control	*p*	Distant Metastases-Free Survival	*p*	Progression-Free Survival	*p*	Overall Survival	*p*
*Age (median 75 years, range 43–85)*								
< 75 years (*n* = 38, 53%)	Median NR	0.3	18 months	0.73	18 months	0.47	Median NR	0.20
≥75 years (*n* = 34, 47%)	Median NR		16 months		14 months		27 months	
*Disease site*								
Juxtabronchial (*n* = 70, 98%)	Median NR	0.6	18 months	0.13	17 months	0.16	Median NR	0.58
Paraesophageal (*n* = 2, 2%)	Median NR		5 months		5 months		Median NR	
*Histotype*								
Adenocarcinoma (*n* = 54, 75%)	Median NR	0.98	16 months	0.48	12 months	0.35	Median NR	0.41
Squamous Cell Carcinoma (*n* = 18, 25%)	Median NR		17 months		17 months		Median NR	
*Timing of Metastatic Dissemination*								
Synchronous (*n* = 47, 65%)	Median NR	0.44	18 months	0.87	18 months	0.67	Median NR	0.17
Metachronous (*n* = 25, 35%)	Median NR		14 months		11 months		Median NR	
*Gross Tumor Volume (median 60 cc, range 7–400 cc)*								
< 60 cc (*n* = 37, 52%)	Median NR	0.24	28 months	0.88	16 months	0.60	Median NR	0.09
≥60 cc (*n* = 35, 48%)	Median NR		16 months		17 months		Median NR	
*Prior Chemotherapy*								
Naïve (*n* = 45, 62%)	Median NR	0.84	44 months	**0.003**	44 months	**0.008**	Median NR	0.15
≥ 1 line (*n* = 27, 38%)	Median NR		7 months		7 months		Median NR	
*BED (median 105, range 75–132 Gy^10^)*								
<75 Gy^10^ (*n* = 67, 93%)	Median NR	**0.02**	6 months	0.24	6 months	**0.036**	Median NR	**<0.001**
≥75 Gy^10^ (*n* = 5, 7%)	Median NR		18 months		17 months		6 months	
*Overall Treatment Time*								
≤1 week (*n* = 13, 18%)	Median NR	0.69	Median NR	0.43	Median NR	0.34	Median NR	0.9
>1 week (*n* = 59, 82%)	Median NR		16 months		16 months		Median NR	

**Table 2:** Summary of toxicity. CTCAE: Common Terminology Criteria for Adverse Events.

CTCAE Grade	Radiation Pneumonitis	Hemoptysis	Radiation Esophagitis	Bronchial Stricture
1	5 (7%)	0 (0%)	1 (2%)	0 (0%)
2	6 (8%)	2 (3%)	3 (5%)	0 (0%)
3	2 (3%)	1 (2%)	0 (0%)	0 (0%)
4	0 (0%)	0 (0%)	0 (0%)	1 (2%)
5	0 (0%)	0 (0%)	1 (2%)	0 (0%)

**Table 3:** Clinical and treatment-related variables and impact on overall and grade ≥3 toxicity. NR: not reached; BED: biologically effective dose. Italic: variable type.

Clinical and Treatment-related Variables	Overall Toxicity		*p*	Grade ≥3 Toxicity		*p*
	No	Yes		No	Yes	
*Age (median 75 years, range 43–85)*						
<75 years	**27 (38%)**	11 (15%)	0.81	37 (51%)	1 (1%)	0.53
≥75 years	**25 (35%)**	9 (12%)		30 (42%)	4 (6%)	
*Disease site*						
Juxtabronchial	51 (71%)	19 (27%)	0.93	65 (90%)	5 (8%)	0.69
Paraesophageal	1 (1%)	1(%)		2 (2%)	0	
*Histotype*						
Adenocarcinoma	36 (50%)	18 (25%)	0.73	50 (69%)	4 (6%)	0.97
Squamous Cell Carcinoma	14 (19%)	4 (6%)		17 (24%)	1 (1%)	
*Timing of Metastatic Dissemination*						
Synchronous	34 (47%)	13 (18%)	0.98	42 (58%)	5 (8%)	0.09
Metachronous	18 (25%)	7 (10%)		25 (34%)	0	
*Gross Tumor Volume (median 60 cc, range 7–400 cc)*						
<60 cc	23 (32%)	14 (19%)	0.07	37 (51%)	3 (5%)	0.51
≥60 cc	27 (37%)	8 (12%)		30 (42%)	2 (2%)	
*Prior Chemotherapy*						
Naive	32 (44%)	13 (18%)	0.79	41 (57%)	4 (6%)	0.29
≥ 1 line	20 (28%)	7 (10%)		27 (37%)	0	
*BED (median 105, range 75–132 Gy^10^)*						
<75 Gy^10^	48 (66%)	19 (27%)	0.91	63 (86%)	4 (6%)	0.65
≥75 Gy^10^	4 (6%)	1 (1%)		5 (8%)	0	
*Overall Treatment Time*						
≤1 week	8 (11%)	5 (8%)	0.53	12 (17%)	1 (1%)	0.75
>1 week	44 (61%)	15 (20%)		56 (78%)	3 (4%)	
